# Resolutions of Respect to Dr. Ellery C. Young

**Published:** 1905-08

**Authors:** 


### RESOLUTIONS OF RESPECT TO DR. ELLERY C. YOUNG.

Whereas, It has pleased Almighty God to remove from our midst on December 6, 1904, at the age of sixty-one, Ellery C. Young, of Leipzig, court dentist to the Grand Duke of Anhalt, be it

*Resolved,* That the American Dental Society of Europe has sustained a serious loss in this, the death of one of its oldest members, who was respected in his community for his uprightness and sterling worth, and who was recognized by all as a superior dentist and a worthy colleague.

*Resolved,* That this resolution be published in the leading dental journals of the United States and a copy be forwarded to the bereaved wife of our friend and colleague, and that this resolution be placed upon record as part of the proceedings of the Society.

WILLIAM A. SPRING,
G. H. WATSON,
W. MITCHELL,
*Committee.*

